# Rhox6 regulates the expression of distinct target genes to mediate mouse PGCLC formation and ESC self-renewal

**DOI:** 10.1186/s13578-023-01096-2

**Published:** 2023-08-08

**Authors:** Xiaofeng Li, Peng Chen, Junxiang Ji, Quanchao Duan, Jianjian Cao, Ru Huang, Shou-Dong Ye

**Affiliations:** Center for Stem Cell and Translational Medicine, School of Life Sciences, Anhui University, Hefei, 230601 Anhui China

**Keywords:** Embryonic stem cells, PGCLCs, Self-renewal, Rhox6, Nanos3, Tbx3

## Abstract

**Background:**

Mouse embryonic stem cells (mESCs) not only retain the property of self-renewal but also have the ability to develop into primordial germ cell-like cells (PGCLCs). However, knowledge about the mechanisms of transcriptional regulation is still limited. *Rhox6*, a member of the homeobox family that is located on the X chromosome, is highly expressed within PGCLCs in vivo and in vitro. However, the detailed effects of *Rhox6* on PGCLC specification and mESC maintenance remain unclear.

**Results:**

In this study, we found that overexpression of *Rhox6* favors the formation of PGCLCs, while depletion of *Rhox6* inhibits the generation of PGCLCs. Mechanistically, *Rhox6* directly induces the expression of *Nanos3* during the specification of PGCLCs. Subsequently, downregulation of *Nanos3* expression is sufficient to decrease the ability of *Rhox6* to induce PGCLC formation. Moreover, we found that depletion of *Rhox6* expression facilitates the self-renewal of mESCs. High-throughput sequencing revealed that suppression of *Rhox6* transcription significantly increases the expression of pluripotency genes. Functional studies further demonstrated that *Rhox6* directly represses the transcription of *Tbx3*. Therefore, knockdown of the expression of the latter impairs the self-renewal of mESCs promoted by *Rhox6* downregulation.

**Conclusions:**

Our study reveals that overexpression of *Rhox6* is beneficial for PGCLC generation through induction of *Nanos3*, while downregulation of *Rhox6* contributes to mESC self-renewal by increasing *Tbx3*. These findings help elucidate the early development of mouse embryos.

**Supplementary Information:**

The online version contains supplementary material available at 10.1186/s13578-023-01096-2.

## Background

Primordial germ cells (PGCs) are the precursor cells of spermatocytes and oocytes. In mice, they were first discovered at the posterior end of the primitive streak in the extraembryonic mesoderm at approximately Embryonic day 6.25 (E6.25). PGC specification then occurs at approximately E7.25 at the base of the incipient allantois [1]. At approximately E10.5, mouse PGCs individually migrate through the hindgut endoderm and mesentery and eventually colonize the embryonic gonads at E11.5 [1]. The normal development of PGCs is a prerequisite for the stable transmission of genetic information between generations. A set of genes has been identified during PGC formation in vivo that includes *Blimp1* and *Prdm14*. The expression of these genes is induced by bone morphogenetic protein 4 (BMP4) produced from the extraembryonic ectoderm [2–5], and their induction in turn upregulates *Tfap2c*, another transcriptional regulator crucial for PGC establishment. The specification of PGCs is a complex process involving inhibition of somatic programming, reacquisition of potential pluripotency, and subsequent genome-wide epigenetic reprogramming [6]. Pluripotent stem cells, such as embryonic stem cells (ESCs) and induced pluripotent stem cells (iPSCs), provide a good model for recapitulating the specialization of PGCs through cytokine exposure in vitro. Briefly, mouse ESCs (mESCs) are first induced to differentiate into epiblast-like cells (EpiLCs) with Activin A and basic fibroblast growth factor (bFGF). Then, EpiLCs are treated with Bmp4, leukemia inhibitory factor (LIF), stem cell factor (SCF) and epidermal growth factor (EGF) in KnockOut Serum Replacement (KSR)-containing medium to further differentiate into PGCLCs [7]. Many important candidate genes, such as *Nanog* [8], *Esrrb* [9], *Otx2* [10], *SETDB1* [11], *Sox17* [12] and *Tfcp2l1* [13], have been identified by using this system. However, further elucidation of the transcription factor circuitry driving the germline program is needed.

In addition to the differentiation process of ESCs, how ESCs maintain their stemness needs to be determined. Originally, mESCs, first established in 1981 [14], were shown to remain in an undifferentiated state on feeder cells, which can be replaced by LIF [15, 16]. LIF binds to LIF receptors and recruits glycoprotein subunit 130 (gp130), activating JAK/STAT3 signaling pathways. Phosphorylated STAT3 stimulates the expression of downstream target genes [17], such as *Klf4* [18], *Gbx2* [19], *Sp5* [20] and *Tfcp2l1* [21], each of which can maintain stem cell self-renewal when overexpressed. In addition to LIF/serum-containing conditions, mESCs can proliferate indefinitely in serum-free conditions in the presence of two small molecules (also known as 2i), CHIR99021 and PD0325901, which inhibit the activity of glycogen synthase kinase-3β (GSK3β) and mitogen-activated protein kinase kinase (MEK), respectively [22]. LIF and 2i have several converged targets [20, 21]. In addition, many self-renewal-promoting factors have been identified [23]. To date, the landscape of self-renewal and pluripotency of mESC maintenance has been described in detail, but the information is not sufficiently comprehensive.

In this study, we found that *Rhox6* was highly expressed in PGCs compared with epiblasts. Functional assays revealed that overexpression of *Rhox6* promotes the formation of PGCLCs but inhibits the self-renewal of mESCs. Through high-throughput sequencing, chromatin immunoprecipitation and gene expression regulation techniques, we demonstrated that *Rhox6* mediates PGCLC specification and mESC maintenance by regulating *Nanos3* and *Tbx3* transcription, respectively. These results provide a new perspective for understanding the regulatory mechanisms of PGC fate decisions and mESC maintenance.

## Results

### Overexpression of *Rhox6* promotes PGCLC formation

**Fig. 1:**
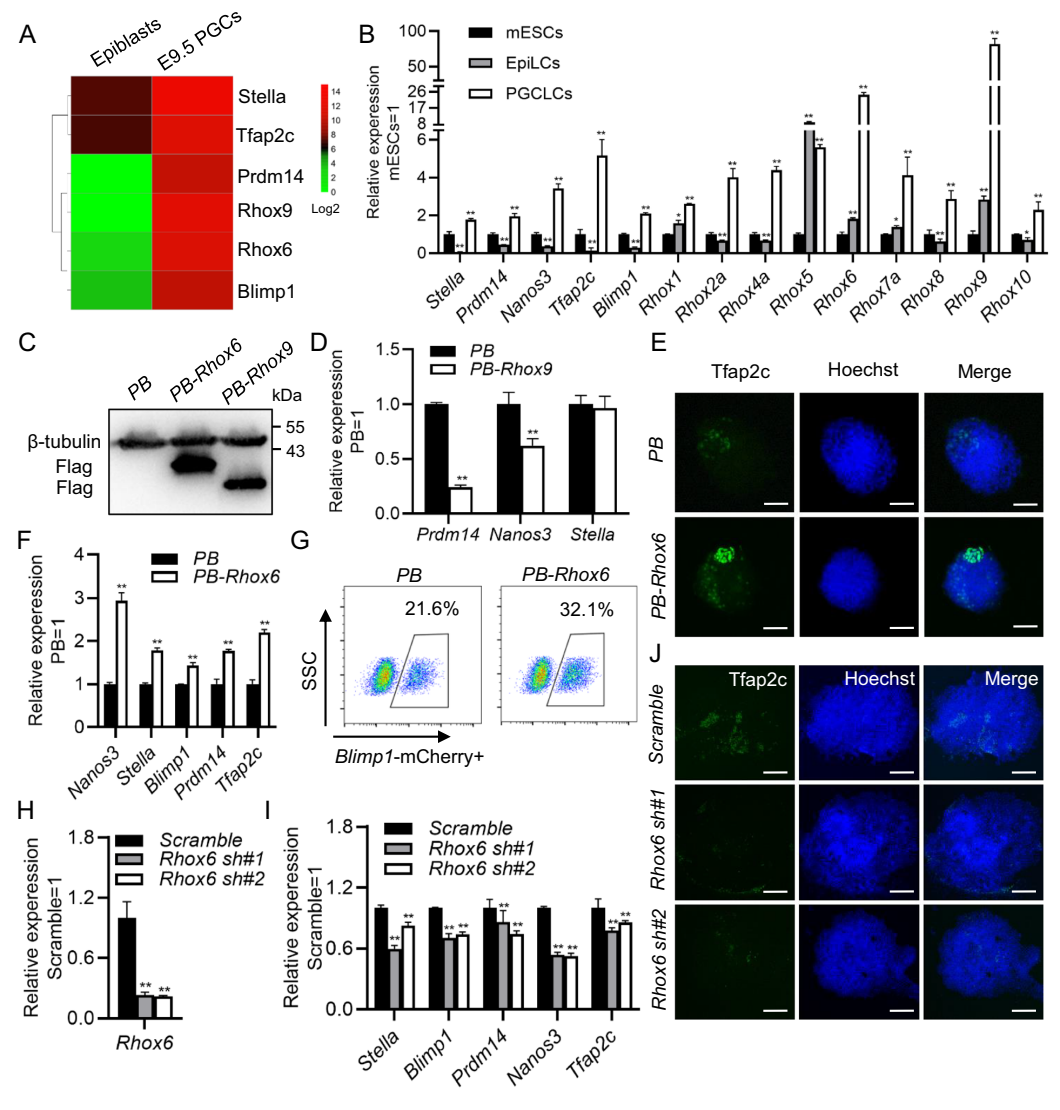
*Rhox6* promotes the differentiation of PGCLCs derived from mESCs. **A** Heatmap analysis of the expression of *Rhox6*, *Rhox9* and PGC marker genes in mouse epiblasts and E9.5 PGCs. **B** qRT-PCR analysis of the expression of PGC marker genes and Rhox family members in mESCs, EpiLCs and PGCLCs. The data are presented as the mean ± SD (N = 3 biological replicates). *P < 0.05, **P < 0.01 versus mESCs, as determined by one-way ANOVA with Sidak’s multiple comparisons test. **C** Western blot analysis of the expression level of Flag in 46C mESCs transfected with *PB*, *PB-Rhox6* or *PB-Rhox9*. β-Tubulin was used as the loading control. **D** qRT-PCR detected the expression of the PGC marker genes *Prdm14*, *Nanos3* and *Stella* in *PB* and *PB-Rhox9* PGCLCs. The data are presented as the mean ± SD (N = 3 biological replicates). **P < 0.01 versus *PB*, as determined by Student’s t test. **E** Immunofluorescence staining of Tfap2c expression in *PB* and *PB-Rhox6* PGCLCs. Scale bar, 100 μM. **F** qRT-PCR analysis of the expression of PGC marker genes in *PB* and *PB-Rhox6* PGCLCs. The data are presented as the mean ± SD (N = 3 biological replicates). **P < 0.01 versus *PB*, as determined by Student’s t test. **G** Flow cytometry analysis of the expression of Blimp1-mCherry in *PB* and *PB-Rhox6* PGCLCs. **H** qRT-PCR analysis of the expression level of *Rhox6* in 46C mESCs infected with *scramble* or *Rhox6* shRNA lentiviruses. The data are presented as the mean ± SD (N = 3 biological replicates). **P < 0.01 versus *scramble*, as determined by one-way ANOVA with Sidak’s multiple comparisons test. **I** qRT-PCR analysis of the expression of PGC marker genes in *scramble* or *Rhox6* shRNA PGCLCs. The data are presented as the mean ± SD (N = 3 biological replicates). **P < 0.01 versus *scramble*, as determined by one-way ANOVA with Sidak’s multiple comparisons test. **J** Immunofluorescence staining of Tfap2c expression in *scramble* and *Rhox6* shRNA PGCLCs. Scale bar, 100 μM

To discover potential candidate genes that may be important for the specification of mouse PGCs, we analyzed the transcriptional data of E9.5 PGCs and epiblasts and found that the homeobox family members *Rhox6* and *Rhox9*, as well as the PGC markers *Prdm14*, *Blimp1*, *Stella* and *Tfap2c*, were highly expressed in PGCs (Fig. 1A). To validate these results in vitro, we differentiated mESCs into EpiLCs with activin A and bFGF for 2 days. Then, the latter were induced to differentiate into PGCLCs in KSR-containing medium with BMP4, LIF, SCF and EGF for 4 days. Quantitative real-time PCR (qRT-PCR) analysis showed that the PGC marker genes *Nanos3*, *Blimp1*, *Tfap2c*, *Stella* and *Prdm14* were upregulated in PGCLCs compared with ESCs and EpiLCs (Fig. 1B). Moreover, all the transcript levels of Rhox family members, including *Rhox1*, *Rhox2a*, *Rhox4a*, *Rhox5*, *Rhox6*, *Rhox7a*, *Rhox8*, *Rhox9* and *Rhox10*, significantly increased (Fig. 1B). Notably, *Rhox6* and *Rhox9* had the highest growth rates, and their expression also gradually increased during the transition from mESCs to EpiLCs (Fig. 1B). These data imply that *Rhox6* and *Rhox9* may promote the fate determination of PGCs.

To validate this hypothesis, we inserted *Flag*-tagged *Rhox6* or *Rhox9* into PiggyBac (PB) plasmids (*PB-Rhox6* or *PB-Rhox9*) and then transfected them with transposons into 46C mESCs. An empty vector was used as a control. Western blot analysis showed that the genes were successfully overexpressed (Fig. 1C and Additional file 1: Fig. S1A). Subsequently, *Rhox6* and *Rhox9* transfectants were induced into PGCLCs. qRT-PCR analysis showed that overexpression of *Rhox9* did not increase the levels of the PGC markers *Prdm14*, *Nanos3* and *Stella* (Fig. 1D). Under the same conditions, the results of immunofluorescence and qRT-PCR revealed that upregulation of *Rhox6* enhanced the expression levels of *Tfap2c*, *Nanos3*, *Stella*, *Blimp1* and *Prdm14* compared with those after transfection with the *PB* control (Fig. 1E, F). Furthermore, we established ESCs with mCherry expression initiated by the *Blimp1* promoter and then transfected them with *PB-Rhox6* [13]. After PGCLC differentiation, flow cytometry screening showed an increase in the number of mCherry-positive cells due to *Rhox6* upregulation (Fig. 1G). These results indicate that upregulation of *Rhox6* favors the generation of PGCLCs.

To examine whether *Rhox6* is necessary for PGCLC production, we designed two mouse *Rhox6* mRNA-specific shRNAs (*Rhox6* shRNAs) with lentiviral systems. *Rhox6* transcripts levels were reduced by approximately 70–80% in 46C mESCs after infection with *Rhox6* shRNA lentivirus compared to the *scramble* control (Fig. 1H and Additional file 1: Fig. S1B). After PGCLC formation, the *Rhox6*-knockdown cells expressed lower levels of the PGC markers *Stella*, *Blimp1*, *Prdm14*, *Nanos3* and *Tfap2c* than the *scramble* control cells (Fig. 1I, J). Then, the endogenous *Rhox6* gene was deleted with the CRISPR/Cas9 system, and the disruption of *Rhox6* alleles was confirmed by genomic DNA sequencing and Western blotting (Additional file 1: Fig. S2A, B). *Rhox6* knockout impaired the efficiency of PGCLC generation (Additional file 1: Fig. S2C). These data suggest that depletion of *Rhox6* limits the formation of PGCLCs.

### *Rhox6* facilitates the specification of PGCLCs by increasing the expression of *Nanos3*

**Fig. 2:**
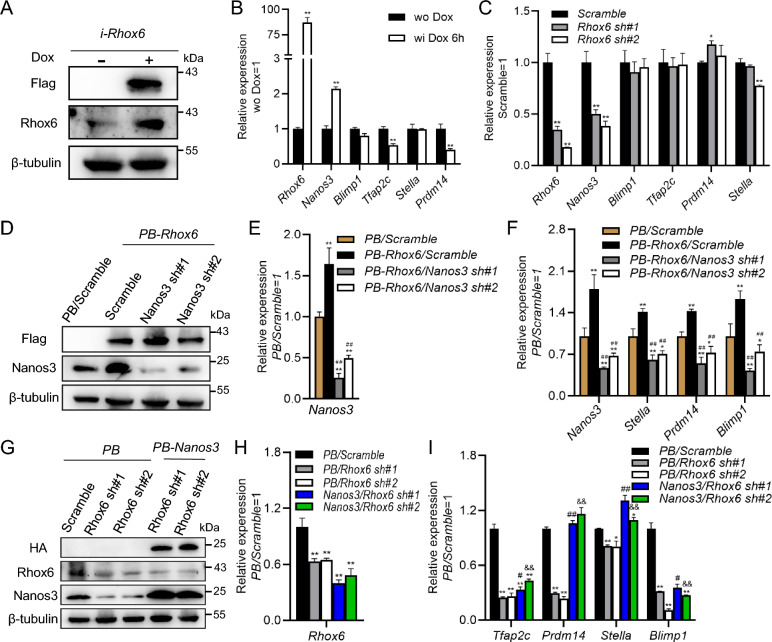
*Rhox6* relies on *Nanos3* to enhance the formation of PGCLCs. **A** Western blot analysis of the expression of Flag and Rhox6 in *i-Rhox6* mESCs, in which *Flag*-tagged *Rhox6* was driven by the Tet-On system, in the presence or absence of 2 μg/ml Dox. **B** qRT-PCR analysis of the expression of *Rhox6* and PGC marker genes in *i-Rhox6* mESCs treated with Dox for 6 h. The data are presented as the mean ± SD (N = 3 biological replicates). **P < 0.01 versus wo Dox, as determined by Student’s t test. Wo, without; wi, with. **C** qRT-PCR analysis of the expression of *Rhox6* and PGC marker genes in 46C mESCs infected with *scramble* or *Rhox6* shRNA lentiviruses. The data are presented as the mean ± SD (N = 3 biological replicates). *P < 0.05, **P < 0.01 versus *scramble*, as determined by one-way ANOVA with Sidak’s multiple comparisons test. **D** Western blot analysis of the expression levels of Flag and Nanos3 in PB and *PB-Rhox6* mESCs infected with *scramble* or *Nanos3* shRNA lentiviruses. **E** qRT-PCR was used to detect the expression level of *Nanos3* in and *PB-Rhox6* mESCs infected with *scramble* or *Nanos3* shRNA lentiviruses. The data are presented as the mean ± SD (N = 3 biological replicates). **P < 0.01 versus *PB/scramble*, as determined by one-way ANOVA with Sidak’s multiple comparisons test. **F** qRT-PCR analysis of the expression of PGC marker genes in PB and *PB-Rhox6* PGCLCs infected with *scramble* or *Nanos3* shRNA lentiviruses. The data are presented as the mean ± SD (N = 3 biological replicates). *P < 0.05, **P < 0.01 versus *PB/scramble*, as determined by one-way ANOVA with Sidak’s multiple comparisons test. **G** Western blot assay of the expression levels of HA, Rhox6 and Nanos3 in *PB* and *PB-Nanos3* mESCs infected with *scramble* or *Rhox6* shRNA lentiviruses. **H** qRT-PCR analysis of the expression levels of *Rhox6* in *PB* and PB-*Nanos3* mESCs infected with *scramble* or *Rhox6* shRNA lentiviruses. The data are presented as the mean ± SD (N = 3 biological replicates). **P < 0.01 versus *PB/scramble*, as determined by one-way ANOVA with Sidak’s multiple comparisons test. **I** qRT-PCR analysis of the expression of PGC genes in *PB* and *PB-Nanos3* PGCLCs infected with *scramble* or *Rhox6* shRNA lentiviruses. The data are presented as the mean ± SD (N = 3 biological replicates). *P < 0.05, **P < 0.01 versus *PB/scramble*, ^#^P < 0.05, ^##^P < 0.01 versus *PB/Rhox6 sh#1*, ^&&^P < 0.01 versus *PB/Rhox6 sh#2*, as determined by one-way ANOVA with Sidak’s multiple comparisons test

To better investigate the mechanism by which *Rhox6* induces PGCLC fate decisions, we constructed a *Flag*-tagged *Rhox6*-inducible cell line with a genome nonintegrated system (*i-Rhox6*), and found that short-term treatment with doxycycline (Dox) for 6 h effectively induced the transient expression of *Rhox6* (Fig. 2A). qRT-PCR showed that compared with no treatment, Dox increased the level of *Nanos3*, one of the markers of PGCs (Fig. 2B). Moreover, downregulation of *Nanos3* was more pronounced in cells infected with *Rhox6* shRNA lentivirus (Fig. 2C).

To further evaluate whether *Nanos3* can mediate the function of *Rhox6* during PGCLC fate decisions, we decreased *Nanos3* transcript levels in *PB*-*Rhox6* cells (Fig. 2D, E). After 4 days of PGCLC differentiation, qRT-PCR analysis results showed that downregulation of *Nanos3* expression reduced the expression of PGC marker genes induced by *PB-Rhox6* compared with the *scramble* control (Fig. 2F). We then enforced the expression of *HA*-tagged *Nanos3* with the PiggyBac vector (*PB-Nanos3*) in *Rhox6* shRNA cells (Fig. 2G). Subsequently, we induced *PB* and *PB-Nanos3* mESCs infected with *Rhox6* shRNA lentivirus to differentiate into PGCLCs (Fig. 2H). qRT-PCR results showed that upregulation of *Nanos3* expression partially alleviated the defects in PGCLC formation caused by downregulation of *Rhox6* expression (Fig. 2I). Collectively, these results suggest that *Rhox6* relies in part on the *Nanos3* gene to mediate the specification of PGCLCs.

### *Nanos3* is a direct target gene of *Rhox6*

**Fig. 3:**
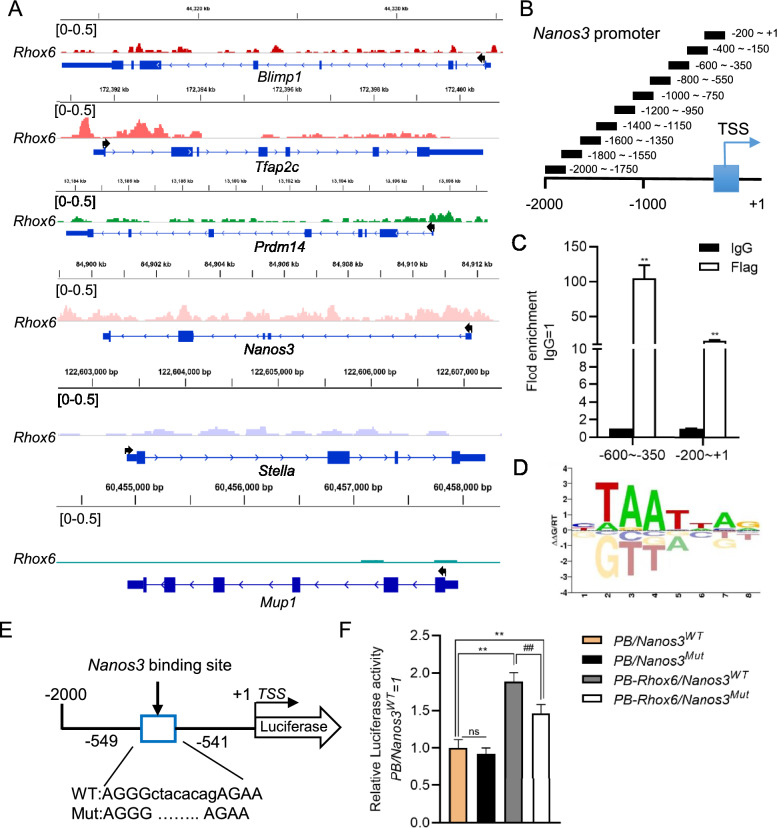
*Nanos3* is a direct target of *Rhox6*. **A** CUT&Tag analysis of the enrichment of *Rhox6* in the promoters of *Blimp1*, *Tfap2c*, *Prdm14*, *Nanos3*, *Stella* and *Mup1*. **B** The *Nanos3* promoter (from − 2000 to + 1) was used as a template, and 10 pairs of qRT-PCR primers were designed. **C** ChIP assays were performed using an anti-Flag antibody. IgG was used as a negative control. The fold enrichment in the indicated regions of the *Nanos3* promoter was measured by qRT-PCR. The data are presented as the mean ± SD (N = 3 biological replicates). **P < 0.01 versus IgG, as determined by Student’s t test. **D** Consensus binding motif of *Rhox6* predicted by the AnimalTFDB database. **E** The binding position and sequence of *Rhox6* in the *Nanos3* promoter and the corresponding deletion mutation sequence. TSS, transcription start site. **F** Luciferase activity analysis of the WT or mutant (Mut) *Nanos3* promoter reporter plasmid-expressing cell lines transfected with PB or *PB-Rhox6*. *PB/Nanos3*^*WT*^ was used as the control for normalization. The data are presented as the mean ± SD (N = 3 biological replicates). *P < 0.05, **P < 0.01 versus *PB/Nanos3*^*WT*^, ^##^P < 0.01 versus *PB-Rhox6/Nanos3*^*WT*^, as determined by one-way ANOVA with Sidak’s multiple comparisons test. ns, not significant

To investigate whether *Rhox6* directly regulates the expression of *Nanos3* in mESCs, we performed a CUT&Tag experiment with an anti-Flag M2 antibody in *PB-Rhox6* cells to pull down the targeted genomic DNA segments, which were then subjected to high-throughput sequencing. The results indicated that there are many *PB-Rhox6* DNA-binding motifs in the *Nanos3* promoter (Fig. 3A). Furthermore, we carried out chromatin immunoprecipitation (ChIP) in *PB-Rhox6* mESCs with an anti-Flag antibody affinity gel and designed ten pairs of qRT-PCR primers with 50 bp repeats between adjacent primers using the *Nanos3* promoter sequence (from − 2000 to + 1) as a template (Fig. 3B). qRT-PCR showed that there was obvious enrichment in the − 600 to − 350 motif (Fig. 3C). Finally, to further illustrate that *Rhox6* is a direct functional activator of *Nanos3* expression, we used the AnimalTFDB database to analyze the binding motifs of *Rhox6* and found one predicted binding site (from − 549 to − 541) in the motif at − 600 to − 350 (Fig. 3D). The promoter fragment with mutated − 549 to − 541 sequences (*Nanos3*^*Mut*^) was cloned. Wild-type (WT) Nanos3 (*Nanos3*^*WT*^) and *Nanos3*^*Mut*^ were then inserted into the pGL3 plasmid to drive the expression of luciferase (Fig. 3E). Thereafter, *PB-Rhox6* and *Renilla* luciferase-expressing plasmids were introduced into 46C mESCs with *pGL3-Nanos3*^*WT*^ or *pGL3-Nanos3*^*Mut*^. After 48 h, these cells were collected and lysed. The results showed that *PB-Rhox6*/*Nanos3*^*WT*^-expressing cells exhibited higher luciferase activity than *PB/Nanos3*^*WT*^- and *PB-Rhox6*/*Nanos3*^*Mut*^*-*expressing cells (Fig. 3F). Together, these results suggest that *Rhox6* binds directly to the promoter of *Nanos3* and stimulates its transcription.

### Knockdown of *Rhox6* favors mESC self-renewal

**Fig. 4:**
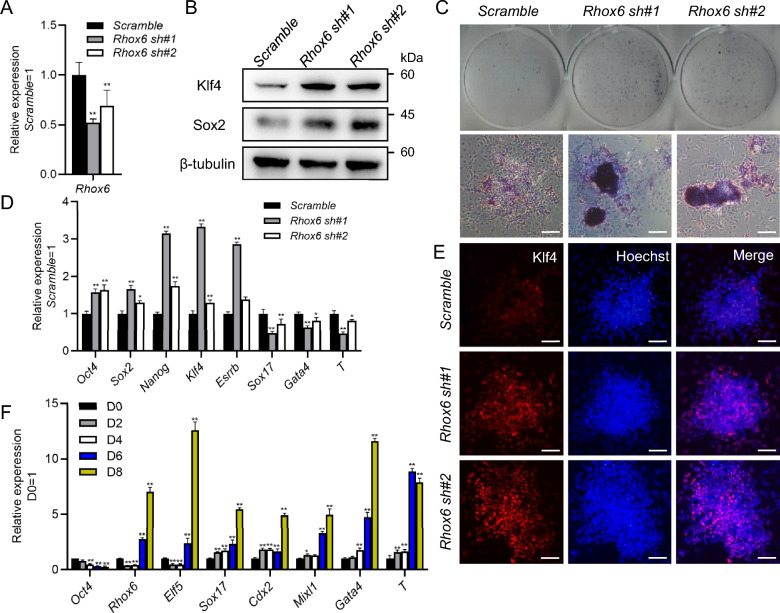
Decreased *Rhox6* levels promote mESC self-renewal. **A** qRT-PCR analysis of the expression level of *Rhox6* in 46C mESCs infected with *scramble* and *Rhox6* shRNA lentiviruses. The data are presented as the mean ± SD (N = 3 biological replicates). **P < 0.01 versus *scramble*, as determined by one-way ANOVA with Sidak’s multiple comparisons test. **B** Western blot analysis of the expression levels of Klf4 and Sox2 in 46C mESCs infected with *scramble* and *Rhox6* shRNA lentiviruses and cultured in the absence of LIF for 7 days. **C** AP staining of *scramble* and *Rhox6* shRNA-expressing mESCs in the absence of LIF for 7 days. Scale bar, 100 μM. **D** qRT-PCR was used to detect the expression of pluripotency and differentiation-related genes in *scramble* and *Rhox6* shRNA mESCs. The data are presented as the mean ± SD (N = 3 biological replicates). *P < 0.05, **P < 0.01 versus *scramble*, as determined by one-way ANOVA with Sidak’s multiple comparisons test. **E** Immunofluorescence staining of Klf4 expression in *scramble* and *Rhox6* shRNA-expressing mESCs. Scale bar, 100 μM. **F** qRT-PCR analysis of the expression of the *Oct4*, *Rhox6*, *Elf5*, *Sox17*, *Cdx2*, *Mixl1*, *Gata4* and* T* genes in mESCs and EBs on different days. The data are presented as the mean ± SD (N = 3 biological replicates). *P < 0.05, **P < 0.01 versus D0, as determined by one-way ANOVA with Sidak’s multiple comparisons test. D0, Day 0

After knockdown of *Rhox6*, we found that mESCs grew better than *scramble* cells and therefore wondered whether downregulation of *Rhox6* facilitates mESC maintenance. To answer this question, we cultured 46C mESCs infected with *scramble* or *Rhox6* shRNA lentiviruses in serum-containing medium without LIF for 7 days. Western blot assays showed that *Rhox6* shRNA cells exhibited higher protein levels of the pluripotency genes Sox2 and Klf4 than *scramble* control cells (Fig. 4A, B). Moreover, alkaline phosphatase (AP) staining and qRT-PCR analysis showed that *Rhox6* shRNA mESCs exhibited higher AP activity and higher levels of the pluripotency genes *Oct4*, *Sox2*, *Klf4*, *Nanog* and *Esrrb* but harbored lower levels of the differentiation-associated genes *Sox17*, *T* and *Gata4* than *scramble* control cells (Fig. 4C–E). Similar results were observed in *Rhox6* knockout cells treated without LIF for 7 days (Additional file 1: Fig. S2D, E). However, overexpression of *Rhox6* was not sufficient to induce mESC differentiation in LIF/serum-containing medium, and these cells had a similar speed of differentiation as *PB* control cells in the absence of LIF (Additional file 1: Fig. S3A–C). Together, these data suggest that low levels of *Rhox6* are able to promote the maintenance of mESC stemness.

To examine the expression pattern of *Rhox6* during mESC differentiation, we suspended 46C mESCs to form embryoid bodies (EBs) to mimic the process of spontaneous differentiation. Cells were collected every 2 days, and qRT-PCR revealed that the expression of *Oct4* decreased, while the transcript levels of the differentiation-related genes *Sox17*, *Cdx2*, *Mixl1*, *Gata4* and *T* gradually increased (Fig. 4F). Notably, the expression of *Rhox6* decreased from Day 2 but increased sharply from Day 6, and the pattern was similar to that of the trophectoderm marker *Elf5* (Fig. 4F), indicating that *Rhox6* may be associated with mESC differentiation.

### Screening of genes downstream of *Rhox6* and associated with mESC self-renewal

**Fig. 5:**
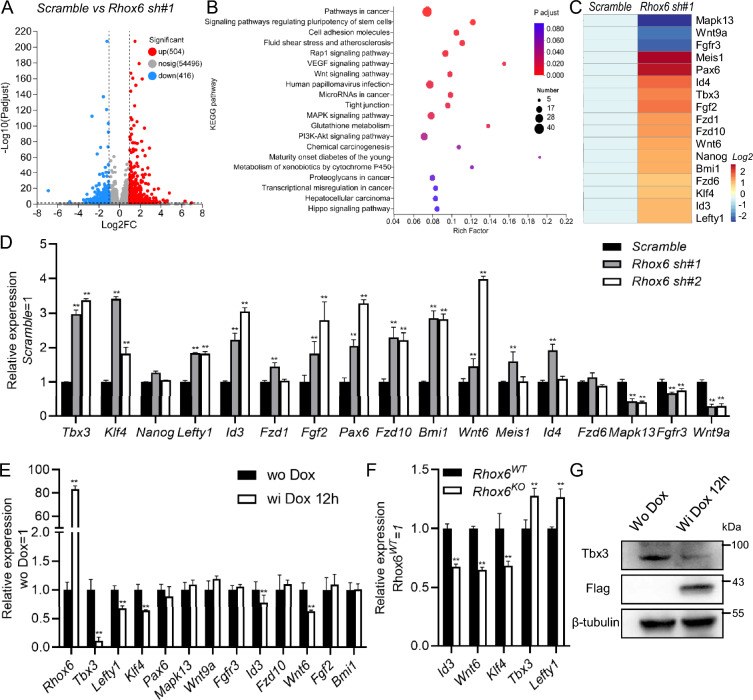
Screening of DEGs regulated by *Rhox6* knockdown. **A** Volcano plot showing DEGs mediated by *Rhox6* downregulation. **B** KEGG analysis of DEGs regulated by *Rhox6* downregulation. **C** Heatmap analysis of the expression of pluripotency-associated genes in DEGs. **D** qRT-PCR analysis of the expression of candidate genes regulated by *Rhox6* knockdown in **C**. The data are presented as the mean ± SD (N = 3 biological replicates). **P < 0.01 versus *scramble*, as determined by one-way ANOVA with Sidak’s multiple comparisons test. **E** qRT-PCR analysis of the expression of *Rhox6* and the indicated genes in *i-Rhox6* mESCs treated with or without Dox for 12 h. The data are presented as the mean ± SD (N = 3 biological replicates). **P < 0.01 versus wo Dox, as determined by Student’s t test. **F** qRT-PCR analysis of the expression of *Id3, Wnt6, Klf4, Tbx3 and Lefty1* in WT and *Rhox6-*knockout mESCs. The data are presented as the mean ± SD (N = 3 biological replicates). **P < 0.01 versus *Rhox6*^*WT*^, as determined by Student’s t test. **G** Western blot analysis of the protein levels of Flag and Tbx3 in *i-Rhox6* mESCs treated with or without Dox for 12 h

We demonstrated that *Rhox6* positively regulated *Nanos3* expression, whereas knockdown of *Nanos3* failed to maintain the undifferentiated state of mESCs in the absence of LIF for 7 days (Additional file 1: Fig. S4A, B). To deeply explore the effect of *Rhox6* on the self-renewal of mESCs, we performed high-throughput sequencing to screen for genes that respond to *Rhox6* knockdown. We found that downregulation of *Rhox6* regulated many differentially expressed genes (DEGs), of which 504 genes were upregulated and 416 genes were downregulated compared with the *scramble* control group (Fig. 5A). To further analyze the biological functions of these DEGs, we performed GO and KEGG signaling pathway analyses (Additional file 1: Fig. S5A–D, Fig. 5B), and found that 17 candidate genes were enriched in signaling pathways regulating pluripotency of stem cells, including *Mapk13*, *Wnt9a*, *Fgfr3*, *Meis1*, *Pax6*, *Id4*, *Tbx3*, *Fgf2*, *Fzd1*, *Fzd10*, *Wnt6*, *Nanog*, *Bmi1*, *Fzd6*, *Klf4*, *Id3*, and *Lefty1* (Fig. 5C). qRT-PCR was performed to verify their expression, and the dynamic expression of most candidates was as expected, except for that of *Nanog*, *Fzd1*, *Meis1*, *Id4* and *Fzd6* (Fig. 5D). Next, we used *Rhox6*-inducible mESCs to confirm the findings. As shown in Fig. 5E, the addition of Dox for 12 h significantly induced *Rhox6* expression but suppressed the transcription of *Tbx3*, *Lefty*, *Klf4*, *Id3* and *Wnt6* (Fig. 5E). These candidate genes were then examined in WT and *Rhox6*-knockout mESCs, and the results showed that depletion of Rhox6 increased *Tbx3* and *Lefty1* transcription (Fig. 5F). However, enforced expression of *Lefty1* did not maintain the stemness of mESCs in the absence of LIF (Additional file 1: Fig. S6A, B). Therefore, we focused on *Tbx3* for the next experiments. Western blot analysis further validated the decreased Tbx3 protein level mediated by *Rhox6* overexpression (Fig. 5G).

### *Tbx3* mediates the function of *Rhox6* in regulating the self-renewal of mESCs

**Fig. 6:**
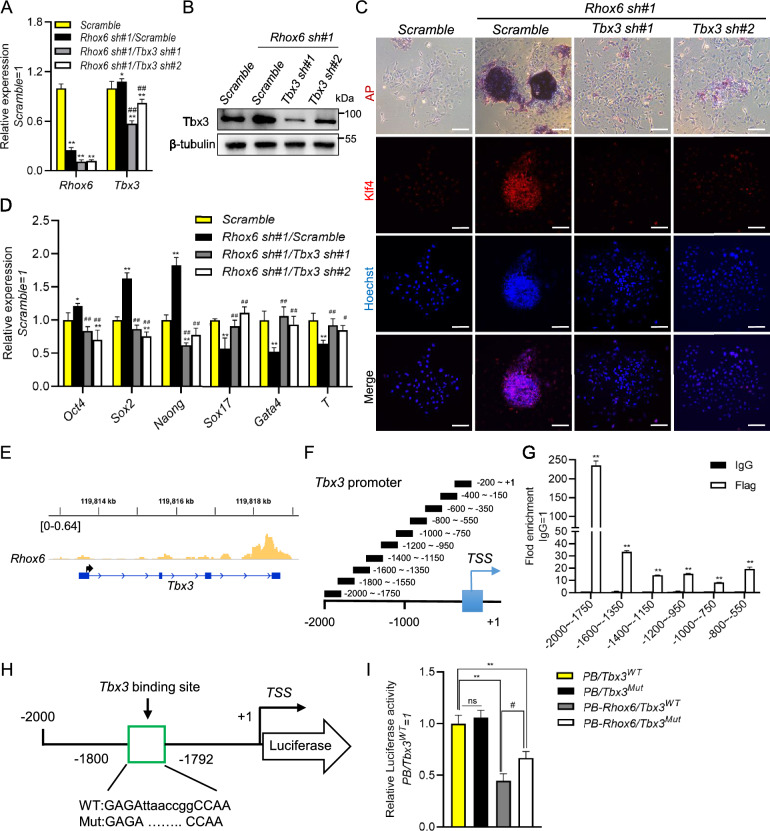
*Tbx3* meditates the self-renewal-promoting effect of *Rhox6* knockdown in mESCs. **A** qRT-PCR analysis of the expression of *Rhox6* and *Tbx3* in *scramble* and *Rhox6* shRNA mESCs infected with *scramble* or *Tbx3* shRNA lentiviruses. The data are presented as the mean ± SD (N = 3 biological replicates). *P < 0.05, **P < 0.01 versus *scramble*, ^##^P < 0.01 versus *Rhox6* sh#1*/scramble*, as determined by one-way ANOVA with Sidak’s multiple comparisons test. **B** Western blot analysis of the protein levels of Tbx3 in *scramble* and *Rhox6* shRNA mESCs infected with scramble or *Tbx3* shRNA lentiviruses. **C** AP staining and immunofluorescence staining of Klf4 in *scramble* and *Rhox6 sh#1* mESCs infected with *scramble* or *Tbx3* shRNA lentiviruses and cultured in serum-containing medium without LIF for 7 days. Scale bar, 100 μM. **D** qRT-PCR analysis of the expression of pluripotency- and differentiation-associated genes. The data are presented as the mean ± SD (N = 3 biological replicates). *P < 0.05, **P < 0.01 versus *scramble*, ^#^P<0.05,  ^##^P<0.01 versus *Rhox6* sh#1/*scramble*,  as determined by one-way ANOVA with Sidak’s multiple comparisons test. **E** CUT&Tag analysis of the binding sites of *Rhox6* on the *Tbx3* promoter. **F** Ten pairs of qRT-PCR primers were designed using the *Tbx3* promoter as the template. **G** A ChIP assay was performed using a Flag antibody. IgG was used as a negative control. The fold enrichment in the indicated regions of the *Tbx3* promoter was measured by qRT-PCR. The data are presented as the mean ± SD (N = 3 biological replicates). **P < 0.01 versus IgG, as determined by Student’s t test. **H** Binding position and sequence of *Rhox6* on the *Tbx3* promoter and the corresponding deletion mutation sequence. **I** Luciferase activity analysis of the WT or mutant (Mut) *Tbx3* promoter reporter plasmid-expressing cell lines transfected with or without *PB-Rhox6*. *PB/Tbx3*^*WT*^ was used as the control for normalization. The data are presented as the mean ± SD (N = 3 biological replicates). *P < 0.05, **P < 0.01 versus *PB/Tbx3*^*WT*^, ^##^P < 0.01 versus *PB-Rhox6/Tbx3*^*WT*^, as determined by one-way ANOVA with Sidak’s multiple comparisons test

To determine whether *Tbx3* can regulate the self-renewal-promoting effect of *Rhox6* shRNA, we constructed two *Tbx3* shRNA plasmids. After packaging, lentiviruses containing *Tbx3* shRNA were used to infect *Rhox6* shRNA mESCs and successfully decreased *Tbx3* transcript levels (Fig. 6A, B). These cell lines were then cultured in serum-containing medium without LIF for 7 days. AP staining and immunofluorescence showed that cells with knockdown of *Rhox6* and *Tbx3* together generated fewer AP-positive colonies and exhibited lower Klf4 expression than *Rhox6/scramble* control cells (Fig. 6C). Moreover, qRT-PCR showed that *Rhox6* and *Tbx3* double-knockdown cells expressed lower levels of the pluripotency markers *Oct4*, *Sox2* and *Nanog* but harbored higher levels of the differentiation-associated genes *Gata4*, *Sox17* and *T* than *Rhox6*-knockdown cells (Fig. 6D). Together, these experimental results indicate that downregulation of *Tbx3* impairs the ability of *Rhox6* knockdown to promote the self-renewal of mESCs.

### The transcription of *Tbx3* is directly suppressed by *Rhox6*

To investigate whether *Rhox6* directly regulates the expression of *Tbx3* in mESCs, we analyzed the CUT&Tag results of *PB-Rhox6* and found many binding sites of *Rhox6* on the promoter of *Tbx3* (Fig. 6E). To confirm this affinity, we designed 10 pairs of primers and performed ChIP (Fig. 6F). qRT-PCR analysis showed that *Rhox6* was significantly enriched in the promoter of *Tbx3*, especially at − 2000 to − 1750 (Fig. 6G). Finally, to validate that *Rhox6* directly suppressed *Tbx3* expression, we combined the results of ChIP and the binding motif of *Rhox6* predicted by the AnimalTFDB database and then inserted WT and mutated *Tbx3* promoter sequences into pGL3 to drive the expression of luciferase (*Tbx3*^*WT*^ and *Tbx3*^*Mut*^) (Fig. 6H). These constructs were transfected into 46C mESCs with *PB-Rhox6* and *Renilla luciferase*-expressing plasmids. After 48 h, these cells were lysed. As shown in Fig. 6I, *PB-Rhox6/Tbx3*^*WT*^ induced lower luciferase activity than *PB/Tbx3*^*WT*^- and *PB-Rhox6/Tbx3*^*Mut*^-expressing cells (Fig. 6I). Overall, these results suggest that *Rhox6* directly inhibits the transcription of *Tbx3*. Notably, the expression of *Tbx3* decreased when mESCs differentiated into EpiLCs and PGCLCs (Additional file 1: Fig. S7A). In addition, overexpression of *Tbx3* had no obvious effect on PGCLC generation (Additional file 1: Fig. S7B–D).

## Discussion

**Fig. 7:**
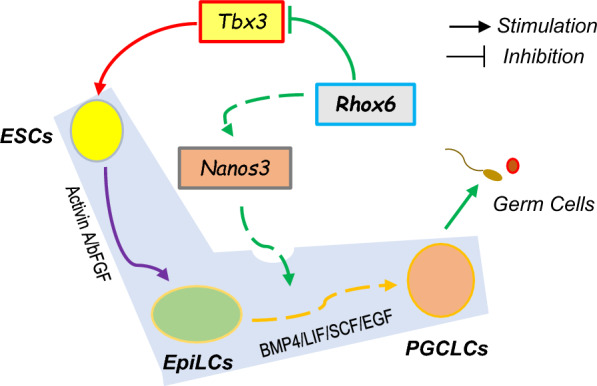
Schematic diagram of the role of *Rhox6* in ESC maintenance and PGCLC specification. Overexpression of *Rhox6* induces *Nanos3* expression to facilitate the formation of PGCLCs derived from EpiLCs. On the other hand, downregulation of *Rhox6* stimulates *Tbx3* expression to favor maintenance of mESC stemness

Analyzing the molecular regulatory network of PGC formation will provide new strategies for future research on reproduction. This study reveals that *Rhox6* promotes the specification of PGCLCs in vitro (Fig. 7). To date, 33 Rhox genes located on the X chromosome have been reported in mice [24], and the top 12 can be divided into three subclusters (α, including Rhox1-4; β, including Rhox 5-9; and γ, including Rhox10-12) [25]. Genes in a subcluster tend to be expressed in a similar manner. Rhox genes are selectively expressed in male and female reproductive tissues, including the testes, epididymis, ovaries, and placenta [24].

*Rhox6* is abundantly expressed in the placenta and postmigratory primordial germ cells [24], highlighting its potential role in regulating PGC fate determination. As expected, loss of *Rhox6* inhibis the generation of mESC-derived PGCLCs [26]. Similar results were also observed in our experiments (Fig. 1H–J and Additional file 1: Fig. S2A–C). However, overexpression of *Rhox6* has been found to have little effect on the specification of male PGCs [26], which is different from our results. This discrepancy may be due to the different methods of mESC differentiation into PGCLCs and the different sex backgrounds of the mESCs [26], as the 46C mESCs used in this study were derived from female mice [26], suggesting that the function of *Rhox6* may be sexually biased, especially in the generation of female primordial germ cells. This result could also be explained by the fact that *Rhox6* is predominantly expressed in embryonic female germ cells. In addition, *Rhox10* is present only in embryonic male germ cells. Transcripts of *Rhox1*, *Rhox6* and *Rhox7* mRNA can be found in fetal ovaries, whereas *Rhox2a*, *Rhox4a*, *Rhox5* and *Rhox9* are detectable in both fetal ovaries and fetal testes [27], suggesting the diverse effects of the Rhox gene on the specification of female and male PGCs. Notably, the *Rhox6* and *Rhox9* genes share approximately 80% identical homeodomains, implying a redundant function between them, whereas overexpression of *Rhox9* has no effect on PGCLC generation (Fig. 1D). It will be of interest to investigate the effect of double knockdown of *Rhox6* and *Rhox9* on PGCLC specification.

Another important finding of our study is the direct regulation between *Rhox6* and *Nanos3*. *Nanos3* belongs to the Nanos family of genes, including *Nanos1*, *Nanos2* and *Nanos3*, which are known for their roles in germ cell development and are conserved in both vertebrates and invertebrates [28]. Nanos genes were first discovered and studied in fruit flies [29, 30]. Their absence blocks PGCs from migrating to undergo gonadal development [31]. Similarly, mice with *Nanos3* knockout have greatly reduced migration of PGCs for reproductive ridge development. *Nanos3* exerts its function in part by preventing apoptosis of PGCs via inhibition of Bax-dependent and Bax-independent mechanisms [32]. In vitro*,* decreased *Nanos3* levels can significantly alter germ cell numbers and the expression patterns of germ cell markers in human ESCs and iPSCs [33, 34]. Our results demonstrate that *Nanos3* is also important for mouse PGCLC formation downstream of *Rhox6*. However, the expression pattern of *Rhox6* and *Nanos3* is not consistent when ESCs differentiate into EpiLCs (Fig. 1B). This phenomenon may be due to the reactivation of two X chromosomes, as one X chromosome is inactivated randomly in mESCs, but all are activated in EpiLCs. Moreover, PGCs undergo X reactivation during migration [35]. As we mentioned above, *Rhox6* expression is closely associated with the state of the X chromosome.

The third important discovery was our demonstration of a mechanistic link between *Rhox6* and *Tbx3* (Fig. 6E–I). *Tbx3*, a member of the T-box gene family, is highly expressed in cell clumps within mouse blastocysts [36]. In line with this, the *Tbx3* level is high in undifferentiated mESCs, but declines when mESCs undergo retinoic acid-induced differentiation [37]. Overexpression of *Tbx3* thus has the ability to bypass the requirement for LIF signaling and maintain ESC pluripotency in vitro by preventing differentiation and increasing self-renewal [37–39]. In contrast, downregulation of *Tbx3* in ESCs causes differentiation [37]. The transcription of *Tbx3* is regulated in part by the phosphatidylinositol-3-OH-kinase-Akt (PI3K) and mitogen-activated protein kinase (MAPK) pathways in mESCs [38]; Tbx3 is also regulated by *Nanog* [40]. At present, our data identify *Rhox6* as a negative modulator of *Tbx3* and show that its expression pattern is different from that of *Tbx3* when ESCs differentiate into cells/aggregates, such as EpiLCs, EBs and PGCs (Fig. 1B, Additional file 1: Fig. S6A) [41]. Consistently, the level of *Rhox6* significantly increased after knockdown of *Oct4* in mESCs [42]. However, overexpression of *Rhox6* is not sufficient to induce mESC differentiation (Additional file 1: Fig. S3A–C). It is likely that LIF produces a strong self-renewal signal that overshadows the differentiation cues induced by *Rhox6* upregulation because when ESCs exit pluripotency, the differentiation signal needs to reach a certain threshold to counteract self-renewal-inducing signals. Notably, *Tbx3* is dispensable for germ cell development [39], but is important for driving mesendodermal and primitive endoderm specification [43, 44]. It is worth discussing whether *Rhox6* participates in these events in the future.

## Conclusions

Our study clarifies the precise role of *Rhox6* in different cellular states. *Rhox6* stimulates *Nanos3* expression to promote PGCLC specification while inhibiting *Tbx3* transcription in undifferentiated mESCs. These results provide new insights into the regulatory network of mESC maintenance and PGC specification. In addition, we reveal the potential value of *Rhox6* in evaluating pluripotency and the role of Rhox6 in germ cell development, which may facilitate our understanding of infertility.

## Methods

### Cell culture

Culture of 46C mouse embryonic stem cells was performed in tissue culture plates coated with 0.1% gelatin. The medium consisted of DMEM (Biological Industries), 15% fetal bovine serum (FBSNE-01061, Ori Cell), 1× Non-essential amino acids (11140050, Gibco), 1× penicillin/streptomycin (15140122, Gibco), 0.1 mM β-mercaptoethanol (M3148, Sigma) and LIF (Made in house).

### Plasmid construction

The coding regions of mouse *Rhox9*, *Rhox6, Nanos3* and *Tbx3* were inserted into PiggyBac transposon vectors (PB) with Flag or HA tags to construct overexpression plasmids. *Rhox6*, *Nanos3* and *Tbx3* shRNA sequences were inserted into the pLKO.1-TRC vector (#10878, Addgene) to construct *Rhox6*, *Nanos3* and *Tbx3* shRNA lentivirus plasmids. The related sequences used are listed in Additional file 1: Tables S1 and S2.

### Cell transfection and virus production

Two micrograms of PB and 2 μg of transposon plasmids were transfected into cells using Hieff Trans Liposomal Transfection Reagent (40802ES03, Yeasen) according to the manufacturer’s instructions. For lentivirus production, 2 μg of pLKO.1, 0.75 μg of VSVG and 1.25 μg of psPAX2 were transfected into 293T cells. After 2 days, the supernatant was collected and used to infect cells. Puromycin and/or blasticidin S HCl was applied to screen the cells.

### Construction of the *Rhox6* knockout cell line

The plasmid pX330-U6-Chimeric_BB-CBh-hSpCas9 (Addgene, #42230) carrying *Rhox6* gDNA was transfected into 46C mESCs with Lipofectamine 3000 (L3000015, Life Technologies). After 48 h of puromycin screening, single colonies were picked and expanded. When the cells grew to a certain density, the genomic DNA was extracted and sent for DNA sequencing. In addition, the disruption of *Rhox6* was further confirmed by Western blotting. The gDNA sequence is CAAGACAGCCGCCAAAGCA.

### qRT-PCR

Total cellular RNA was extracted using the MolPure Cell/Tissue Total RNA Kit (19221ES50, Yeasen), and cDNA was synthesized from 1 μg of total RNA with the HiScript III All-in-one RT SuperMix Perfect for qPCR Kit (R333-01, Vazyme). Finally, qRT-PCR was carried out with qPCR SYBR Green Master Mix (Without ROX) reagent (Q121-02, Vazyme) in a PikoReal Real-Time PCR Machine. The relative expression was determined by the 2-ΔCq method and normalized to the expression of mouse Rpl19. The primers used are listed in Additional file 1: Table S3.

### Western blot

Cells were lysed with cold RIPA buffer (P0013B, Beyotime Biotechnology, China) supplemented with protease inhibitors. The extracted proteins were separated on 10% PAGE gels and electrically transferred to a PVDF membrane. The primary antibodies were Flag (1:1000, GNI4110-FG-S, GNI), HA (1:1000, GNI4110-HA-S, GNI, 1:1000), β-tubulin (1:2000, 200608, ZENBIO), Sox2 (1:1000, 66411-1-Ig, Proteintech), Klf4 (1:1000, R381633, ZENBIO), Tbx3 (1:1000, R25871, ZENBIO), Rhox6 (1:100, PA5-68779, ThermoFisher) and Nanos3 (1:500, ab70001, Abcam).

### Alkaline phosphatase staining

Cells were fixed with 4% paraformaldehyde for 2 min and washed twice with PBS. After incubation in AP staining reagent (C3206, Beyotime Biotechnology, China) for 30 min at room temperature according to the manufacturer’s instructions, cells were observed under a Leica DMI8 microscope.

### Immunofluorescence staining

Cells were washed with PBS three times and then fixed with 4% paraformaldehyde for 20 min at room temperature. After incubation in blocking buffer (PBS containing 5% BSA and 0.2% Triton X-100) for 2 h, the cells were placed in the diluent of primary antibody at 4 °C overnight. The antibodies were Klf4 (1:500, R381633, ZENBIO) and Tfap2c (sc12762, 1:100, Santa Cruz). After three washes with PBS, the cells were then incubated with a fluorescent secondary antibody and Hoechst 33342 (H3570, Invitrogen, 1:10,000) for 1 h at 37 °C in the dark. The cells were photographed under a Leica DMI8 microscope.

### Flow cytometry

PGCLCs expressing Blimp1-mCherry were digested into single cells with Solase solution (RP01021, Nuwacell, China) and then resuspended in 500 μl of cold DPBS. The fluorescence intensity of cells was analyzed by CytoFLEX flow cytometry (Beckman).

### CUT&Tag assay

*PB-Rhox6* mESCs were resuspended in cold DPBS and counted with a cell counter. CUT&Tag was performed with a CUT&Tag Kit (TD903, Vazyme, China). A Flag antibody (1:100, F1804, Sigma-Aldrich) was used to pull down the DNA fragments. A DNA library was established using the TruePrep Index Kit V2 for Illumina kit (TD202, Vazyme). High-throughput sequencing was used to analyze the sequence information in detail. The screen shots of peak enrichment were analyzed by IGV (version 2.12.3).

### ChIP assay

ChIP experiments were performed by following the instructions of a ChIP Analysis Kit (P2078, Beyotime Biotechnology). A Flag antibody was used for immunoprecipitation, and IgG was used as a negative control. The enrichment of ChIP was verified by qRT-PCR. The primer sequences and locations within the promoter regions of *Nanos3* and *Tbx3* are listed in Additional file 1: Tables S4 and S5.

### Luciferase assay

The promoter sequences of *Nanos3* (− 800 to + 1) and *Tbx3* (− 2000 to − 1400) were cloned into pGL3 plasmids (*pGL3-Nanos3*, *pGL3-Tbx3*). WT and mutant *pGL3-Nanos3* or *pGL3-Tbx3* plasmids were cotransfected into 46C mouse ESCs with *PB-Rhox6* and Renilla-luciferase plasmids. After 48 h, luciferase activity was detected using the TransDetect Double-Luciferase Reporter Assay Kit (FR201, TransGen Biotech, China).

### PGCLC induction

First, 46C ESCs (3 × 10^5^) were seeded in plates coated with fibronectin (16.7 μl/ml, F1141-5MG, Sigma) and cultured in serum-free N2B27 medium with 20 ng/ml Activin A (C678, Novoprotein, China), 12 ng/ml bFGF (C044, Novoprotein, China) and 1% KSR (10828028, Invitrogen) to induce EpiLCs. Two days later, 2 × 10^5^ EpiLCs were exposed to PGCLC-inductive medium containing BMP4 (500 ng/ml, 315-27-10, Peprotech), LIF (1000 U/ml, Millipore), SCF (100 ng/ml, AF-250-03, Peprotech), EGF (50 ng/ml, AF-10015, Peprotech), 15% KSR and GMEM medium for 4 days to induce PGCLCs.

### Statistical analysis

The number of biological replicates is stated in each legend. All data are reported as the mean ± SD. Data were visualized with GraphPad Prism 8. Two paired Student’s t test or one-way ANOVA with Sidak’s multiple comparisons test was used to determine the significance of differences in the following comparisons. p < 0.05 indicated statistical significance.

### Supplementary Information

**Additional file 1: Figure S1.** Analysis of the expression of endogenous *Rhox6* in *Rhox6-*overexpressing and *Rhox6*-knockdown mESCs. Related to Fig. 1. **Figure S2.** Effect of *Rhox6* knockout on mouse PGCLC specification and ESC maintenance. Related to Figs. 1 and 4. **Figure S3.** Overexpression of *Rhox6* has little effect on the self-renewal of mESCs. Related to Fig. 4. **Figure S4.** Effect of *Nanos3* knockdown on the self-renewal of mESCs. Related to Fig. 5. **Figure S5.** Analysis of DEGs regulated by *Rhox6* knockdown. Related to Fig. 5. **Figure S6.** Overexpression of *Lefty1* fails to maintain the undifferentiated state of mESCs. Related to Fig. 5. **Figure S7.** *Tbx3* has little impact on PGCLC specification. Related to Fig. 6. **Table S1.** List of primers used for gene overexpression. Related to Experimental procedures. **Table S2.** List of shRNA sequence used for gene knockdown. Related to Experimental procedures. **Table S3.** List of primers used for qRT-PCR analysis. Related to Experimental procedures. **Table S4.** List of primers used for ChIP-qRT-PCR analysis of *Nanos3*. Related to Experimental procedures. **Table S5.** List of primers used for ChIP-qRT-PCR analysis of *Tbx3*. Related to Experimental procedures.

## Data Availability

The datasets used and/or analyzed during the current study are available from the corresponding author on reasonable request. Our transcriptome sequencing data has been deposited in the GEO database with the accession number GSE222172.

## Abbreviations

mESCs: Mouse embryonic stem cells
PGCLCs: Primordial germ cell-like cells
EpiLCs: Epiblast like cells
Rhox6: Reproductive homeobox 6
Nanos3: Nanos homolog 3
Tbx3: T-box 3
PB: PiggyBac
Dox: Doxcycline
qRT-PCR: Quantitative real time PCR
