# Supplementary material for: Rhox6 regulates the expression of distinct target genes to mediate mouse PGCLC formation and ESC self-renewal
Source: Cell Biosci. 2023 Aug 8;13:145. doi: 10.1186/s13578-023-01096-2 (PMC10408072; doi:10.1186/s13578-023-01096-2)
Supplement: Supplementary file 1 — Additional file 1: Figure S1. Analysis of the expression of endogenous Rhox6 in Rhox6-overexpressing and Rhox6-knockdown mESCs. Related to Fig. 1. Figure S2. Effect of Rhox6 knockout on mouse PGCLC specification and ESC maintenance. Related to Figs. 1 and 4. Figure S3. Overexpression of Rhox6 has little effect on the self-renewal of mESCs. Related to Fig. 4. Figure S4. Effect of Nanos3 knockdown on the self-renewal of mESCs. Related to Fig. 5. Figure S5. Analysis of DEGs regulated by Rhox6 knockdown. Related to Fig. 5. Figure S6. Overexpression of Lefty1 fails to maintain the undifferentiated state of mESCs. Related to Fig. 5. Figure S7. Tbx3 has little impact on PGCLC specification. Related to Fig. 6. Table S1. List of primers used for gene overexpression. Related to Experimental procedures. Table S2. List of shRNA sequence used for gene knockdown. Related to Experimental procedures. Table S3. List of primers used for qRT-PCR analysis. Related to Experimental procedures. Table S4. List of primers used for ChIP-qRT-PCR analysis of Nanos3. Related to Experimental procedures. Table S5. List of primers used for ChIP-qRT-PCR analysis of Tbx3. Related to Experimental procedures. [file 13578_2023_1096_MOESM1_ESM.pdf]

Additional file 1

Figure S1

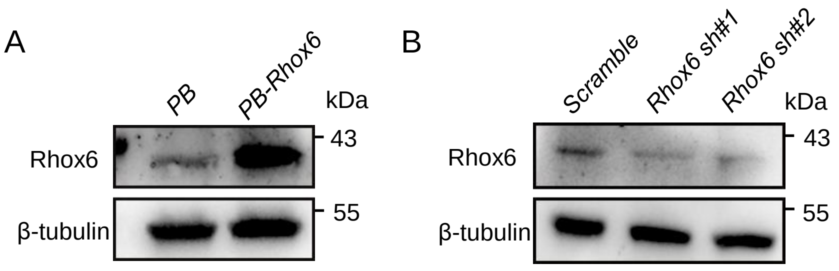

Figure S1. Analysis of the expression of endogenous *Rhox6* in *Rhox6*-overexpressing and *Rhox6*-knockdown mESCs. Related to Figure 1.

(A) Western blot analysis of *Rhox6* expression in mESCs transfected with *PB* or *PB-Rhox6*.

(B) Western blot analysis of *Rhox6* expression in mESCs infected with *scramble* or *Rhox6 shRNA* lentivirus.

Figure S2

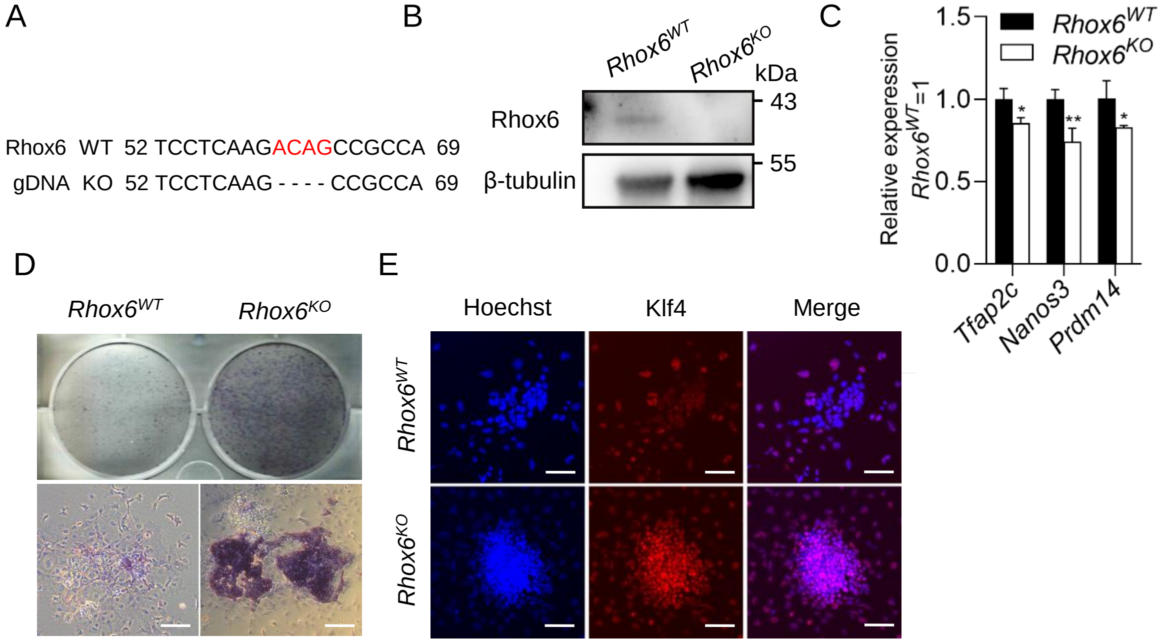

Figure S2. Effect of *Rhox6* knockout on mouse PGCLC specification and ESCs. Related to Figure 1 and 4.

(A) Disruption of mouse *Rhox6* by CRISPR/Cas9 was verified by sequencing of genomic DNA. The knockout (KO) DNA sequence is indicated in red.

(B) Western blot analysis of the protein levels of *Rhox6* in *Rhox6*^{WT} and *Rhox6*^{KO} mESCs. WT, wild type.

(C) qRT–PCR analysis of the expression of PGC markers in *Rhox6*^{WT} and *Rhox6*^{KO} PGCLCs. The data are presented as the mean±SD (N = 3 biological replicates). *P < 0.05, **P < 0.01 versus *Rhox6*^{WT}, as determined by Student's t test.

(D) AP staining of *Rhox6*^{WT} and *Rhox6*^{KO} mESCs seeded in serum-containing medium without LIF for 7 days. Scale bars: 100 μm.

(E) Immunostaining of Klf4 in *Rhox6*^{WT} and *Rhox6*^{KO} mESCs in the absence of LIF for 7 days. Scale bars: 100 μm.

Figure S3

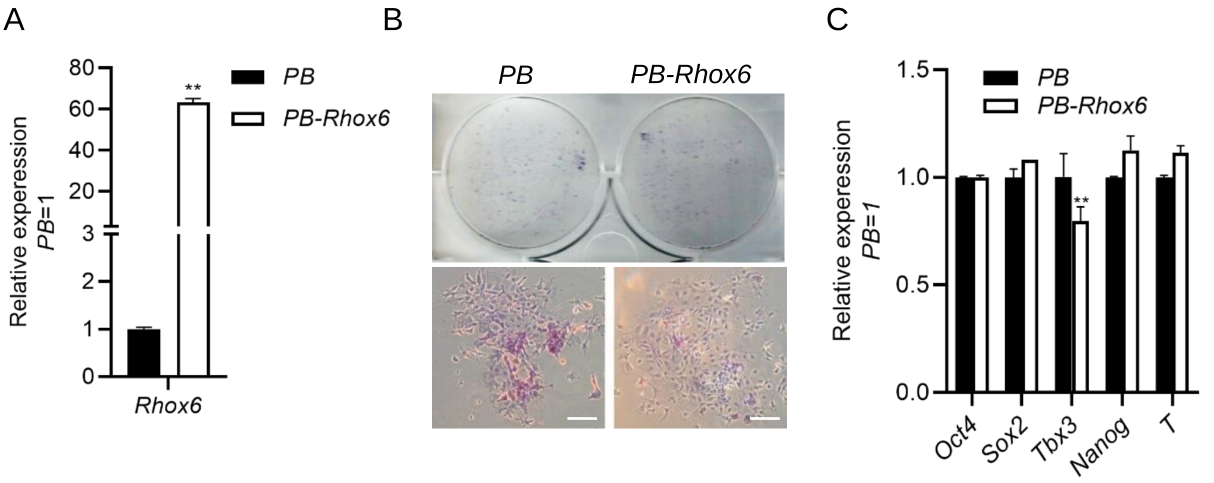

Figure S3. Overexpression of *Rhox6* has little effect on the self-renewal of mESCs. Related to Figure 4.

(A) qRT–PCR analysis of *Rhox6* expression in mESCs transfected with *PB* or *PB-Rhox6*. The data are presented as the mean±SD (N = 3 biological replicates). **P < 0.01 versus *PB*, as determined by Student's t test.

(B) AP staining of *PB* and *PB-Rhox6* mESCs cultured in serum-containing medium in the absence of LIF for 7 days. Scale bar, 100 μM.

(C) qRT–PCR analysis of the gene expression of *Oct4, Sox2, Tbx3, Nanog* and *T* in *PB* and *PB-Rhox6* mESCs treated without LIF for 7 days. The data are presented as the mean±SD (N = 3 biological replicates). **P < 0.01 versus *PB*, as determined by Student's t test.

Figure S4

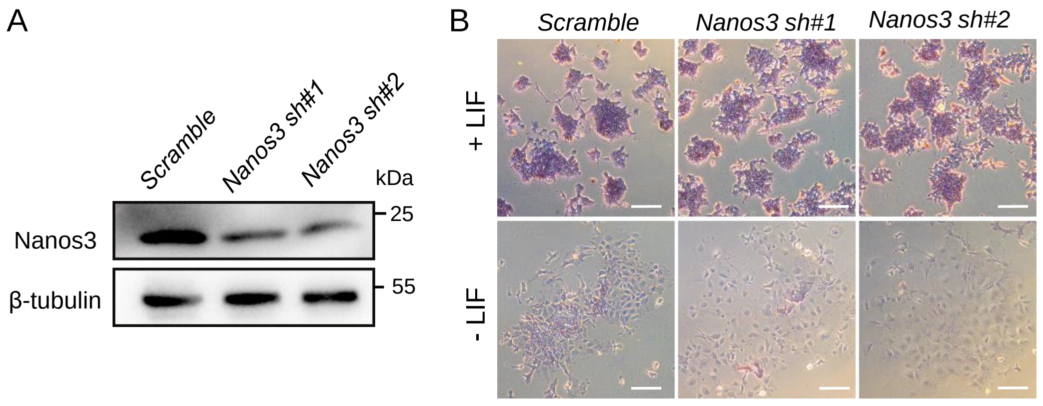

Figure S4. Effect of *Nanos3* knockdown on the self-renewal of mESCs. Related to Figure 5.

(A) Western blot analysis of the expression of *Nanos3* in *scramble* and *Nanos3* shRNA mESCs.

(B) AP staining of *PB* or *Nanos3 shRNA* mESCs grown in serum-containing medium in the absence or presence of LIF for 7 days. Scale bar, 100 μM.

Figure S5

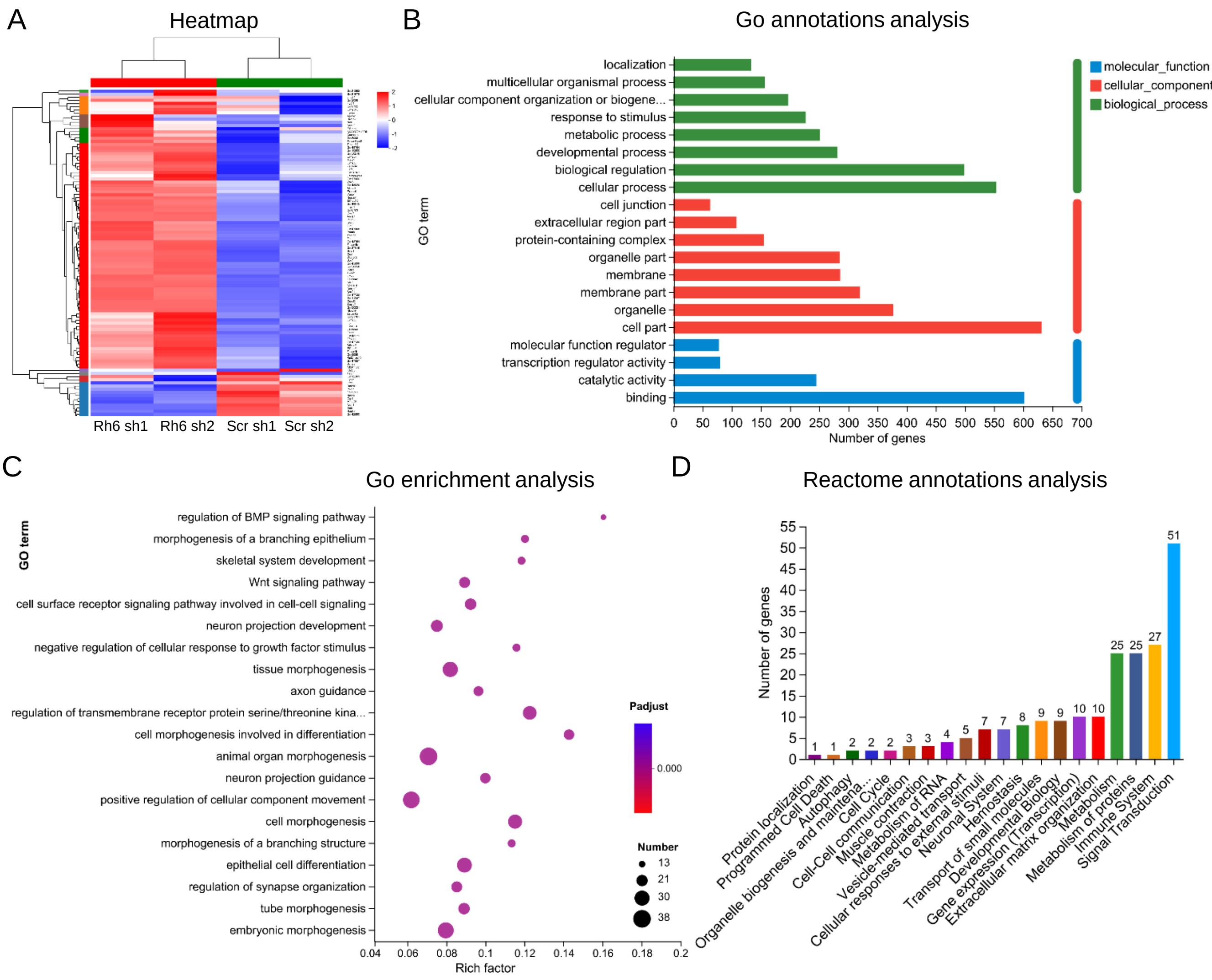

Figure S5. Analysis of DEGs regulated by *Rhox6* knockdown. Related to Figure 5.

(A) Heatmap showing the expression of DEGs regulated by *Rhox6* downregulation in 46C mESCs.

(B) GO annotation analysis of DEGs regulated by *Rhox6* knockdown.

(C) GO enrichment analysis of DEGs regulated by *Rhox6* knockdown.

(D) Reactome annotation analysis of DEGs regulated by *Rhox6* knockdown.

Figure S6

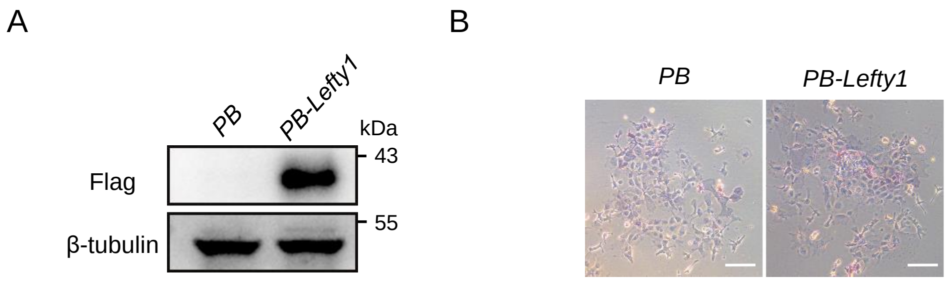

Figure S6. Overexpression of *Lefty1* fails to maintain the undifferentiated state of mESCs. Related to Figure 5.

(A) Western blot analysis of the expression of Flag in mESCs transfected with or without flag-tagged *Lefty1* (*PB-Lefty1)*

(B) AP staining of *PB* or *PB-Lefty1* mESCs grown in serum-containing medium without LIF for 7 days. Scale bar, 100 μM.

Figure S7

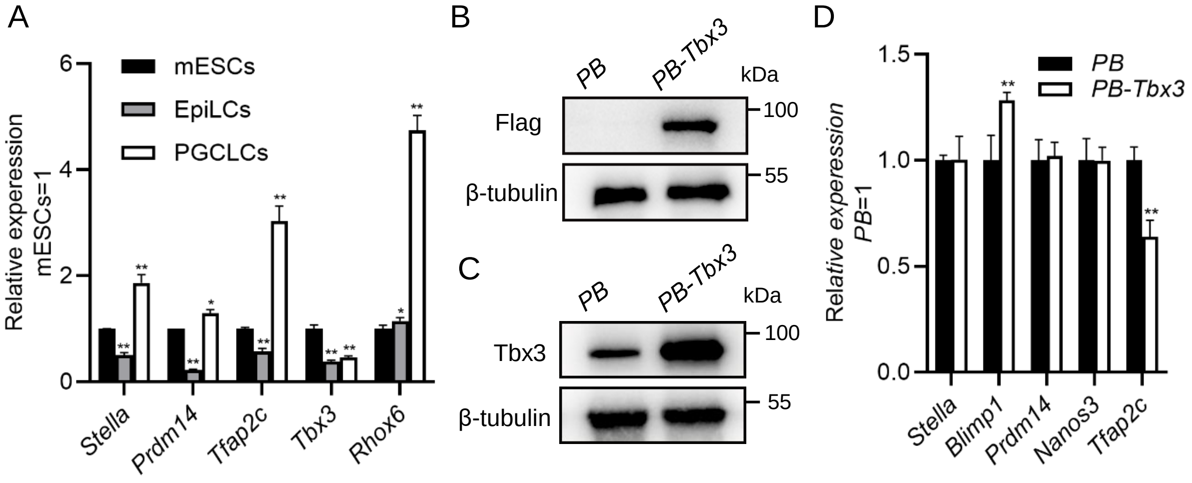

Figure S7. *Tbx3* has little impact on PGCLC specification. Related to Figure 6.

(A) qRT–PCR analysis of the expression of *Tbx3*, *Rhox6* and the PGC marker genes *Stella*, *Prdm14* and *Tfap2c* in mESCs, EpiLCs and PGCLCs. The data are presented as the mean±SD (N = 3 biological replicates). *P<0.05, **P<0.01 versus mESCs, as determined by one-way ANOVA with Sidak's multiple comparisons test.

(B) Western blot analysis of the expression level of Flag in 46C mESCs transfected with *PB* or *PB-Tbx3*.

(C) Western blo analysis of the expression level of Tbx3 in 46C mESCs transfected with *PB* or *PB-Tbx3*.

(D) qRT–PCR analysis of the expression of PGC marker genes in *PB* and *PB-Tbx3* PGCLCs. The data are presented as the mean±SD (N = 3 biological replicates). **P< 0.01 versus *PB*, as determined by Student's t test.

Table S1. List of primers used for gene overexpression.
Related to Experimental procedures.

Symbol	Forward sequence (5'-3')	Reverse sequence (5'-3')
Rhox9	ATGGAGACTCCTCAAGACAGCCG	TCAGGGAGAGTTGTTCTCTGTAATC
Rhox6	ATGGAAACTCCTCAAGACAGCCG	TCAGGGAGAGTCGCTCTGGGG
Nanos3	ATGGGGACTTTCAATCTTTGGACAG	CTAGGCCGTAGTGGAGGGACAGCAG
Tbx3	ATGAGCCTCTCCATGAGAGATCCGG	TTAAGGGGACCCGCTGCAAGACCTG
Lefty1	ATGCCATTCCTGTGGCTCTGCT	CTATGGCTGCAGCCTCCTGGGTATG

Table S2. List of shRNA sequence used for gene knockdown.
Related to Experimental procedures.

Symbol	Forward sequence (5'-3')
Rhox6 sh#1	CCAGTCCTGATAGCATCAGAA
Rhox6 sh#2	GAAACAGGAGAGTGCTGATGT
Nanos3 sh#1	GCTGGAGACTGGACCTTGCTT
Nanos3 sh#2	CAGCTCCTGAACGCTTATGTT
Tbx3 sh#1	GCGAATGTTCCCTCCGTTTAA
Tbx3 sh#2	GAAACAGAATTCATCGCCGTT

Table S3. List of primers used for qRT-PCR analysis.
Related to Experimental procedures.

Symbol	Forward sequence (5'-3')	Reverse sequence (5'-3')
Rpl19	GACGGAAGGGCAGGCATATG	TGTGGATGTGCTCCATGAGG
Stella	GTCGGTGCTGAAAGACCCTA	TCCCGTTCAAACTCATTTCC
Blimp1	AGCATGACCTGACATTGACACC	CTCAACACTCTCATGTAAGAGGC
Nanos3	CACTACGGCCTAGGAGCTTGG	TGATCGCTGACAAGACTGTGG
Tfap2c	GGGCTTTTCTCTCTTGGCTGGT	TCCACACGTCACCCACACAA
Prdm14	AAGCCTTTGCATCTCATGCT	AGGAAGCCTTTCCCACAAAT
Rhox1	CTTCTTTTCCAGCGCACTCA	TTGGCTTCACACACACACAG
Rhox2a	AATGTAAAGCGTGTGTCCGG	GCTCTTGTAGTTGCAGCACA
Rhox4a	GGCCTAAGTCAGTGTGGGTA	TCTTTCCTCTGCACGGATGA
Rhox5	AGAAGAAACAGGAGGAGGGC	ATCTCACTCCACGACAAGCA
Rhox6	AGAGGCAGCCAGTCCTGATA	CTGGGGTAGCGAGTCTCTT
Rhox7a	AAGGAAAACAGCTGGCAAGG	CTTCAGAGAGGTGGAGTGGG
Rhox8	TACCAAGTTCCAGCTCCAGG	ACACCTATCCATCTCGCGAG
Rhox9	GCTGGGAACTATCTGGCTCA	CTGTAATCGGTGGCAGTTCG
Rhox10	AATGCAAGGTGAAGGCTTGG	CAAGATGTTGCTGGCACACA
Oct4	AGAGGGAACCTCCTCTGAGC	TTCATGTCCTGGGACTCCTC
Sox2	CACAACTCGGAGATCAGCAA	CTCCGGGAAGCGTGTACTTA
Nanog	GTCTGATTCAGGGCTCAGCA	AAGGCTTCCAGATGCGTTCA
Klf4	CCAAAGAGGGGAAGAAGGTC	CTGTGTGAGTTCGCAGGTGT
Esrrb	TACCTGAACCTGCCGATTTC	ATCTGGTCCCCAAGTGTCAG
Sox17	TAAAGGTGAAAGGCGAGGTG	CTTAGCTCTGCGTTGTGCAG

Symbol	Forward sequence（5'-3'）	Reverse sequence（5'-3'）
Gata4	TCTCACTATGGGCACAGCAG	GGGACAGCTTCAGAGCAGAC
T	CCCTGCACATTACACACCAC	CCCCTTCATACATCGGAGAA
Elf5	GGACCGATCTGTTCAGCAAT	GCTGCCTCAATGAACTCCTC
Cdx2	AAGACAAATACCGGGTGGTG	CCAGCTCACTTTTCCTCCTG
Mixl1	TTGAATTGAACCCTGTTGTCCC	GAAACCCGTTCTCCCATCCACC
Tbx3	CAGCTCACACTGCAGTCCAT	GAGACAGCAGGAGAGGATGC
Lefty1	AGCTCAAGGCAATTGTGACC	TCATCTCTGAGGCGACACAC
Id3	TTAGCCAGGTGGAAATCCTG	TCAGTGGCAAAAGCTCCTCT
Fzd1	ATCGTCATCGCCTGCTACTT	AGACGGTAGTCTCCCCCTGT
Fgf2	GGCTGCTGGCTTCTAAGTGT	TATGGCCTTCTGTCCAGGTC
Pax6	AGTGAATGGGCGGAGTTATG	ACTTGGACGGGAACTGACAC
Fzd10	CAAGACACCTGACTGCCTGA	TTCCCAAGGTGAGGTTTTTG
Bmi1	CCAGGGCTTTTCAAAAATGA	GCATCACAGTCATTGCTGCT
Wnt6	CTTCGGGGATGAGAAGTCAA	AAAGCCCATGGCACTTACAC
Meis1	AAGGTGATGGCTTGGACAAC	ACGCTTTTTGTGACGCTTTT
Id4	ACTCACCCTGCTTTGCTGAG	AGAATGCTGTCACCCTGCTT
Fzd6	TGTTGGGCTGTCTCTCCTCT	TCTCCCAGGTGATCCTGTTC
Mapk13	CATTGGGCTTCTGGATGTCT	CCCAAAGTCCAGGATCTTCA
Fgfr3	ACCGAGTCTACACCCACCAG	TGAGGATGCGGTCTAAATCC
Wnt9a	TGCTTTCCTCTACGCCATCT	CCCAGGAACTCCTTGACAAA

Table S4. List of primers used for ChIP-qRT-PCR analysis of *Nanos3*.
Related to Experimental procedures.

Location from UCSC	Forward sequence (5'-3')	Reverse sequence (5'-3')
-2000~-1750	TAAATTGGGAGACTCTGGGG	AAGAGCCTTCAAGCACAGGA
-1800~-1550	ACCTGCTGGGGTATGCCAGT	ACAAGGAGGACCCTGTTAAA
-1600~-1350	CTGCCATGTTCTCGCAGCAT	TGAGTTCCAGGACAGCCAGG
-1400~-1150	GGTTTTTTAAGGCAGGGTTT	ACAATTACAGGCAGGGGCAG
-1200~-950	CCACTCTGGAGACTCCTAGG	GGGAGGGCAGTCCAGCTCCT
-1000~-750	ACTCTGCCCCCAACCTTGAA	ACAACTGGGTCTCAAAGCTC
-800~-550	TCCAGGACATGAAAGTCTGT	CCTGGCTGTCCTGGAACTCA
-600~-350	TCTGAGTTCAAGGCCAGCCT	GGCAGGGGGTGGGAGGTGGG
-400~-150	AGAGAAAGAAACTCAAGGCC	CATGTGGCCTTGAGTTTCTT
-200~+1	TCAGTCTGCATCTGGCTACT	AGAAACCCAACCTGGCTCCA

Table S5. List of primers used for ChIP-qRT-PCR analysis of *Tbx3*.
Related to Experimental procedures.

Location from UCSC	Forward sequence (5'-3')	Reverse sequence (5'-3')
-2000~-1750	GGCCAGATTATTTGCATGCA	CCAGAGCCGCCCTAGAACCC
-1800~-1550	ATTAACCGGCCAATGTCGGT	GAGAAAGAGGGAGAGAAACG
-1600~-1350	AGCCACCGCCTTTGGTACCA	TAGGGCAGAACATTTGAGGC
-1400~-1150	ATCAAAACACGTAAGACTGG	AAGACCTTTGCTTGGAAAGG
-1200~-950	TAGGCCCGGAAACCCAGCCT	TTACCCCAGATGTCGGGAGA
-1000~-750	CTCCCCACCCTAGGATGAGC	CACCTCCGGGCCAGCTCGGT
-800~-550	CAGCTCTCCAAGCCCAGAGC	CCTGGCCCAGTCTACGCTCG
-600~-350	AATACATTTTTTAAAAAAAA	CGCCCGGGTGCTTCGGATTC
-400~-150	ATTGGTGGGATCCCCTGCGC	CTACAGTTCAAGCCCGCGGC
-200~+1	GAAGAAGCTGCAGATCCGCA	GTCTGAAAGGCCTCAGCCAT
